# Genomic, transcriptomic and epigenomic sequencing data of the B-cell leukemia cell line REH

**DOI:** 10.1186/s13104-023-06537-2

**Published:** 2023-10-10

**Authors:** Mariya Lysenkova Wiklander, Elin Övernäs, Johanna Lagensjö, Amanda Raine, Anna Petri, Ann-Christin Wiman, Jon Ramsell, Yanara Marincevic-Zuniga, Henrik Gezelius, Tom Martin, Ignas Bunikis, Sara Ekberg, Rikard Erlandsson, Pontus Larsson, Mai-Britt Mosbech, Susana Häggqvist, Susanne Hellstedt Kerje, Lars Feuk, Adam Ameur, Ulrika Liljedahl, Jessica Nordlund

**Affiliations:** 1 Department of Medical Sciences and Science for Life Laboratory, Uppsala University, Box 1432, Uppsala, SE-751 44 Sweden; 2 Department of Immunology, Genetics and Pathology and Science for Life Laboratory, Uppsala University, Uppsala, Sweden

**Keywords:** Next-generation sequencing, Long-read, Short-read, Multi-omics, Genomics, Transcriptomics, Epigenomics, B-ALL, Cell line, Digital karyotype

## Abstract

**Objectives:**

The aim of this data paper is to describe a collection of 33 genomic, transcriptomic and epigenomic sequencing datasets of the B-cell acute lymphoblastic leukemia (ALL) cell line REH. REH is one of the most frequently used cell lines for functional studies of pediatric ALL, and these data provide a multi-faceted characterization of its molecular features. The datasets described herein, generated with short- and long-read sequencing technologies, can both provide insights into the complex aberrant karyotype of REH, and be used as reference datasets for sequencing data quality assessment or for methods development.

**Data description:**

This paper describes 33 datasets corresponding to 867 gigabases of raw sequencing data generated from the REH cell line. These datasets include five different approaches for whole genome sequencing (WGS) on four sequencing platforms, two RNA sequencing (RNA-seq) techniques on two different sequencing platforms, DNA methylation sequencing, and single-cell ATAC-sequencing.

## Objective

Human cell lines are commonly used by researchers as accessible models of disease [1, 2]. The REH cell line, derived from a fifteen-year old female patient at relapse, is frequently used in the study of ALL, the most common cancer in children [3, 4], as well as for method development [5]. At the same time, next-generation sequencing has become an invaluable tool for cancer research [6, 7], while long-read technology increasingly offers novel insights into complex oncological aberrations [8, 9]. Therefore, a multi-faceted dataset encompassing the genomics, transcriptomics and epigenomics of a cell line such as REH can be a valuable resource for leukemia researchers. Likewise, developers of bioinformatic analysis software stand to benefit from the availability of publicly available reference datasets [10, 11].

A subset of the datasets in this project were used for downstream analysis with the purpose of cataloging the structural variants and fusion genes of the REH cell line [12]. For this project, mapping was performed to the human reference genome GRCh38. Additionally, the long-read WGS datasets were subjected to de-novo assembly.

Here, we present the raw sequencing reads as well as assemblies and mapped BAM files in order to make the data available to the research community.

## Data description

The project consists of 33 sequencing datasets generated from the ALL cell line REH, which was obtained from DSMZ (ACC 22) and cultured according to the supplier’s specifications (see Supplemental Methods) [13, 14]. The cell line’s authenticity was confirmed by karyotyping [12] and STR analysis [15]. The datasets are divided into nine library types. Of the seven library types using DNA as input, five are whole genome sequencing (WGS) methods producing genomic data [16–34], one is a method producing chromatin accessibility data (single-cell ATAC-seq) [35–39], and one is a whole genome methylome sequencing method producing epigenomic data (EM-seq) [40, 41]. The WGS methods include Illumina TruSeq DNA PCR-Free, PacBio SMRT, Oxford Nanopore (ONT), MGISEQ stLFR and linked-read WGS (10x Genomics), while RNA was used as input to two RNA-seq methods, Illumina TruSeq Stranded Total RNA and PacBio IsoSeq [42–48]. The datasets include raw sequencing reads in FASTA and FASTQ formats, reference-mapped BAM files, and de-novo assemblies (Table 1).

### Genomic datasets

The genomic data consists of short- and long-read sequencing datasets, including de-novo assemblies, providing a combination of generous coverage and contiguity that allows for the in-depth analysis of the genomic variation present in this cell line. Included are FASTQ files from the short-read WGS sequencing of two lanes prepared with the Illumina TruSeq DNA PCR-Free kit and sequenced on the HiSeq X sequencer with PE150 read-length, as well as a BAM file of the reads mapped to human reference genome GRCh38.

Long-read WGS datasets include FASTQ files generated from a CLR library and a HiFi library sequenced on the PacBio Sequel II, as well as six ONT libraries prepared with three different kits using DNA selected to varying sizes and sequenced on the PromethION 24. BAM files mapping reads generated from both PacBio libraries and the ONT ultralong library to GRCh38 are included, as are three de-novo assemblies generated from these reads using hifiasm and flye.

Additionally, there are FASTQ files from one WGS library prepared with BGI’s MGIEasy stLFR kit and sequenced on the MGISEQ-2000RS, as well as from two linked-read WGS libraries prepared using the 10x Genomics Gemcode kit and sequenced on the Illumina HiSeq 2500.

### Chromatin accessibility datasets

Single-cell ATAC-seq enables the selective sequencing of chromatin-accessible genomic regions, allowing for the determination of chromatin accessibility profiles on a cellular level. A library was prepared using the Chromium Single Cell ATAC Reagent Kit from 10X Genomics and sequenced on an SP flowcell on an Illumina NovaSeq 6000 instrument. FASTQ data, plus a BAM file mapping this data to GRCh38, are included among the datasets.

### Epigenomic datasets

Methylome analysis of the REH cell line can be performed using the epigenomic data sets, which identify 5-mC or 5-hmC modifications to DNA. Two such libraries were prepared with 10 ng and 100 ng input DNA using the NEBNext enzymatic methyl-seq kit (EM-seq). The libraries were sequenced on an Illumina NovaSeq 6000 on an S4 flowcell.

### Transcriptomic datasets

The datasets include both short-read and long-read transcriptomic data, allowing insight into gene expression and aberrations such as fusion genes, as well as detailed transcript splicing information. The RNA-seq datasets include FASTQ files from the short-read sequencing of two lanes prepared with the Illumina TruSeq Stranded Total RNA kit and sequenced PE-100 on a NovaSeq 6000 instrument, as well as a BAM file of these reads mapped to GRCh38. The long-read RNA-seq data consists of two IsoSeq libraries, with a varying bead ratio used to generate one library with standard-length transcripts and one library with full-length transcripts. An additional dataset containing resulting FLNC reads and a BAM file mapping them to GRCh38 is included for each of the IsoSeq libraries.

**Table 1:** Overview of data files/data sets

Label	Name of data file/data set	File types (file extension)	Accession
Data file 1	Overview of REH sequencing datasets	PNG (.png)	Figshare: 10.6084/m9.figshare.23966340 [13]
Data file 2	Supplemental Methods	PDF (.pdf)	Figshare: 10.6084/M9.FIGSHARE.22643065 [14]
Data file 3	Short Tandem Repeat Analysis of the REH cell line	PDF (.pdf)	Figshare: 10.6084/m9.figshare.24131670 [15]
Dataset 1	REH_WGS_Illumina_HiSeqX_TruSeqDnaPcrFree_1	FASTQ (fastq.gz)	SRA: https://identifiers.org/insdc.sra:SRR10882610 [16]
Dataset 2	REH_WGS_Illumina_HiSeqX_TruSeqDnaPcrFree_2	FASTQ (fastq.gz)	SRA: https://identifiers.org/insdc.sra:SRR10882609 [17]
Dataset 3	REH_WGS_Illumina_HiSeqX_TruSeqDnaPcrFree_hg38	BAM (.bam)	SRA: https://identifiers.org/insdc.sra:SRR23704824 [18]
Dataset 4	REH_WGS_PacBio_SequelII_SMRT_CLR	FASTQ (fastq.gz)	SRA: https://identifiers.org/insdc.sra:SRR22805329 [19]
Dataset 5	REH_WGS_PacBio_SequelII_SMRT_HiFi	FASTQ (fastq.gz)	SRA: https://identifiers.org/insdc.sra:SRR19123265 [20]
Dataset 6	REH_WGS_PacBio_SMRT_hg38	BAM (.bam)	SRA: https://identifiers.org/insdc.sra:SRR23704823 [21]
Dataset 7	REH_WGS_PacBio_SequelII_SMRT_denovo_hifiasm	FASTA (.fa)	SRA: https://identifiers.org/insdc.sra:SRR23704827 [22]
Dataset 8	REH_WGS_ONT_PromethION_10kb	FASTQ (fastq.gz)	SRA: https://identifiers.org/insdc.sra:SRR22730978 [23]
Dataset 9	REH_WGS_ONT_PromethION_20kb	FASTQ (fastq.gz)	SRA: https://identifiers.org/insdc.sra:SRR22444744 [24]
Dataset 10	REH_WGS_ONT_PromethION_30kb	FASTQ (fastq.gz)	SRA: https://identifiers.org/insdc.sra:SRR23054498 [25]
Dataset 11	REH_WGS_ONT_PromethION_60kb_SRE	FASTQ (fastq.gz)	SRA: https://identifiers.org/insdc.sra:SRR22444743 [26]
Dataset 12	REH_WGS_ONT_PromethION_SRE	FASTQ (fastq.gz)	SRA: https://identifiers.org/insdc.sra:SRR22444742 [27]
Dataset 13	REH_WGS_ONT_PromethION_Ultralong	FASTQ (fastq.gz)	SRA: https://identifiers.org/insdc.sra:SRR21147769 [28]
Dataset 14	REH_WGS_ONT_PromethION_Ultralong_hg38	BAM (.bam)	SRA: https://identifiers.org/insdc.sra:SRR23704822 [29]
Dataset 15	REH_WGS_ONT_PromethION_Ultralong_denovo_flye_medaka	FASTA (.fasta)	SRA: https://identifiers.org/insdc.sra:SRR23704826 [30]
Dataset 16	REH_WGS_ONT_PromethION_Ultralong_PacBio_SMRT_denovo_flye_racon	FASTA (.fasta)	SRA: https://identifiers.org/insdc.sra:SRR23704825 [31]
Dataset 17	REH_WGS_MGISEQ_2000RS_stLFR	FASTQ (fastq.gz)	SRA: https://identifiers.org/insdc.sra:SRR18907774 [32]
Dataset 18	REH_WGS_Illumina_HiSeq2500_10x_Gemcode_HW_hg37	BAM (.bam)	SRA: https://identifiers.org/insdc.sra:SRR10902121 [33]
Dataset 19	REH_WGS_Illumina_HiSeq2500_10x_Gemcode_NW_hg37	BAM (.bam)	SRA: https://identifiers.org/insdc.sra:SRR10902122 [34]
Dataset 20	REH_ScAtacSeq_Illumina_NovaSeq6000_10x_Chromium_1	FASTQ (fastq.gz)	SRA: https://identifiers.org/insdc.sra:SRR22320001 [35]
Dataset 21	REH_ScAtacSeq_Illumina_NovaSeq6000_10x_Chromium_2	FASTQ (fastq.gz)	SRA: https://identifiers.org/insdc.sra:SRR22320000 [36]
Dataset 22	REH_ScAtacSeq_Illumina_NovaSeq6000_10x_Chromium_3	FASTQ (fastq.gz)	SRA: https://identifiers.org/insdc.sra:SRR22319999 [37]
Dataset 23	REH_ScAtacSeq_Illumina_NovaSeq6000_10x_Chromium_4	FASTQ (fastq.gz)	SRA: https://identifiers.org/insdc.sra:SRR22319998 [38]
Dataset 24	REH_ScAtacSeq_Illumina_NovaSeq6000_10x_Chromium_hg38	BAM (.bam)	SRA: https://identifiers.org/insdc.sra:SRR10907069 [39]
Dataset 25	REH_EMSeq_Illumina_NovaSeq6000_NEBNext_100ng	FASTQ (fastq.gz)	SRA: https://identifiers.org/insdc.sra:SRR23020114 [40]
Dataset 26	REH_EMSeq_Illumina_NovaSeq6000_NEBNext_10ng	FASTQ (fastq.gz)	SRA: https://identifiers.org/insdc.sra:SRR23020113 [41]
Dataset 27	REH_RnaSeq_Illumina_NovaSeq6000_TruSeqStrandedTotalRna_1	FASTQ (fastq.gz)	SRA: https://identifiers.org/insdc.sra:SRR10882846 [42]
Dataset 28	REH_RnaSeq_Illumina_NovaSeq6000_TruSeqStrandedTotalRna_2	FASTQ (fastq.gz)	SRA: https://identifiers.org/insdc.sra:SRR10882845 [43]
Dataset 29	REH_RnaSeq_Illumina_NovaSeq6000_TruSeqStrandedTotalRna_hg38	BAM (.bam)	SRA: https://identifiers.org/insdc.sra:SRR23704830 [44]
Dataset 30	REH_RnaSeq_PacBio_SequelII_IsoSeq_standard	FASTQ (fastq.gz)	SRA: https://identifiers.org/insdc.sra:SRR22729869 [45]
Dataset 31	REH_RnaSeq_PacBio_SequelII_IsoSeq_long	FASTQ (fastq.gz)	SRA: https://identifiers.org/insdc.sra:SRR22729868 [46]
Dataset 32	REH_RnaSeq_PacBio_SequelII_IsoSeq_standard_hg38	BAM (.bam)	SRA: https://identifiers.org/insdc.sra:SRR23704829 [47]
Dataset 33	REH_RnaSeq_PacBio_SequelII_IsoSeq_long_hg38	BAM (.bam)	SRA: https://identifiers.org/insdc.sra:SRR23704828 [48]

## Limitations

- The 10x Genomics Gemcode linked-read sequencing technology is discontinued.
- The MGISEQ WGS data was sequenced to low (~ 10x) sequencing depth.
- The REH cells used to generate the datasets herein were obtained from a single source. Given that cell lines may undergo alterations during proliferation, leading to genetic heterogeneity within the cell population, these data may not serve as a universal reference for all REH cultures.

## Data Availability

The data described in this Data note can be freely and openly accessed on SRA under BioProject PRJNA600820 [16–48]. Supplemental methods are available at 10.6084/M9.FIGSHARE.22643065 [14].
